# Poverty and Cataract—A Deeper Look at a Complex Issue

**DOI:** 10.1371/journal.pmed.0050245

**Published:** 2008-12-16

**Authors:** Susan Lewallen

## Abstract

Susan Lewallen discusses a new study that shows an association between visual impairment from cataract and poverty in three low-income countries.

It is hardly debatable that poverty and poor health, however one measures either of these broad concepts, often exist together. Blindness, the most important manifestation of poor eye health, is no exception. World Health Organization estimates show higher rates of visual impairment in poorer countries, with few exceptions [1], and a recent large population-based survey in Pakistan found an association between blindness and poverty within-country [2].

The most common cause of blindness worldwide is cataract. This condition mostly affects older people, rich and poor alike, but there is an effective surgical cure. Therefore, while cataract blindness is relatively rare in countries where good quality eye surgery is available to the majority, cataract still accounts for half or more of the visual impairment in countries where people have limited access to eye care [1] .

## A New Case-Control Study

In a new study published in this issue of *PLoS Medicine*, Hannah Kuper and colleagues sought to show an association between visual impairment from cataract and poverty in three low-income countries: Kenya, Bangladesh, and the Philippines [3]. The study had a case-control design: cases were people with visual impairment due to cataract and controls were those with no visual impairment. Since many elderly people depend on their families [4], the authors measured household wealth; to increase the validity of this difficult measurement, they used three different indicators of poverty. The researchers found that cases with visual impairment due to cataract were poorer than controls in all three countries studied. They conclude that there is a significant relationship between poverty and visual impairment from cataract.

**Linked Research Article**

This Perspective discusses the following new study published in *PLoS Medicine*:

Kuper H, Polack S, Eusebio C, Mathenge W, Wadud Z, et al. (2008) A case-control study to assess the relationship between poverty and visual impairment from cataract in Kenya, The Philippines, and Bangladesh. PLoS Med 5(12): e244. doi:10.1371/journal.pmed.0050244

Hannah Kuper and colleagues report a population-based case-control study conducted in three countries that found an association between poverty and visual impairment from cataract.

There are a few methodological issues that deserve consideration. The researchers tried to match cases and controls for age and sex, but there were still significantly older people among cases, especially in Kenya. This mismatch is a concern since households with extremely elderly members may be more impoverished than others. The authors used logistic regression to try to control for this mismatch, as well as to control for other potential confounders. However, they did not take into account the fact that controls were significantly more likely than cases to be literate, to be better educated, and to have jobs other than field work. This limitation of their study is important, since these factors could certainly confound an association between poverty and cataract visual impairment.

For the sake of discussion, however, let's make the assumption that there is an association between visual impairment from cataract and poverty. What does the association mean? Are there implications for programs seeking to eliminate blindness or hoping to reduce poverty?

First, the question of whether poverty causes cataract blindness, or cataract blindness causes poverty, cannot be answered from a case-control study. It is easy to imagine that lack of money keeps the poor from accessing cataract surgery. On the other hand, it is possible that visual impairment restricts earning potential, either for the affected person or for other members of the household who have to take care of the patient. However, trying to reduce this association to a simple cause and effect relationship is overly simplistic. Both poverty and the process involved in getting cataract surgery are complex issues.

## Unraveling the Complex Web

It is useful to try to unravel the complex web of poverty and blindness by considering the social determinants of health. These refer to the myriad factors in a society that determine health, of which economic status is only one. Cataract visual impairment provides an excellent example of the concept of social determinants. Consider what must happen for individuals with visual impairment from cataract to receive surgery.

First, they must be aware of the problem, recognizing that vision loss in old age is not “normal” and that it may be fixed. They must know where to go, how much time and money are required, and what happens during and after treatment. It is interesting that, in all three studies cited by the authors to support the idea that cost is the major barrier to cataract surgery, lack of “awareness of surgery” was actually more commonly given as a reason than cost [5–7]. Awareness is determined by factors within the health system (e.g., are the services well-known to communities and first-line health workers?) as well as factors such as patient or family education level. It is likely that people who are literate or have access to sophisticated technology (e.g., television or cell phones) are more aware of small decreases in vision than people without these advantages. In Kuper and colleagues' study, the cases were not only poorer than controls, they were significantly less likely to be literate and educated. Could these factors have affected their awareness of cataract and cataract surgery?

Once patients with cataract are aware of the problem and possible solutions, the next issue they face is access to services. Success here depends on many factors, including the existence of convenient services, social support to reach them, and, of course, money for transport and service fees.

Finally comes the issue of acceptance. Although one might assume that an elderly person with cataract would happily accept surgery if it were provided free and made available, surprisingly often this is not the case. Acceptance is influenced by a wide range of personal and cultural beliefs, including a patient's belief that surgery will restore vision [8]. Unfortunately, in resource-poor settings, cataract surgery is not always successful; for example, only 50%–70% of eyes in the populations studied by Kuper and colleagues achieved normal visual acuity after surgery [5–7]. When we studied Tanzanian patients with cataract who said they were too poor to pay for surgery and interviewed them at their homes, we found there were often multiple reasons why they did not want eye surgery. Only 22% took advantage of offers of fee waivers and transportation [9]. Education, again, is probably involved in the complex issue of acceptance of surgery.

For another example of the importance of the social determinants of eye health, consider trachoma, another cause of blindness associated with poverty. Studies have shown significant decreases in trachoma in communities in the absence of specific trachoma control programs and without any measurable increases in income of the population [10,11]. Better access to water and perhaps increased understanding of hygiene may have been critical factors.

## Conclusion

So there are many factors involved in whether an individual's cataract blindness is cured, of which the economic status of the household is only one. Focusing too narrowly on the monetary costs (direct or indirect) of cataract surgery may lead us to miss other critical social determinants that keep people blind. Strategies that address the multiple complex social and cultural issues involved in restoring vision ought to be the most successful approaches in the long run—and will have the added value of addressing factors that influence health and welfare in general.
